# The surprising role of marital status on sport second-screening: demographic influences during the 2022 world cup viewing in Israel

**DOI:** 10.3389/fspor.2024.1329364

**Published:** 2024-04-08

**Authors:** Yair Galily, Tal Samuel-Azran, Tal Laor

**Affiliations:** ^1^ Sammy Ofer School of Communications at Reichman University, Herzliya, Israel; ^2^ School of Communications at Ariel University, Ariel, Israel

**Keywords:** sport, Israel, social networks, media, soccer

## Abstract

To better understand what characterizes those who use a second screen while watching sport, the study examine a variety of demographic factors influencing browsing device trends before, during (“second screen”), and after sports games. It does so by utilizing survey data from Israeli viewers of the 2022 World Cup using a convenience sample (*N* = 242). In line with our hypotheses, those with higher education and higher reported income were more likely to browse devices for information around and during games. Against our hypothesis, young adults were less likely to engage in browsing before, during and after the games, possibly because they tend to watch games with friends or in public places. Divorced and single individuals are more likely to engage in multi-platform browsing and second-screening during sport games vs. married participants, who tended to watch the games with friends or in public places. The results are the first to indicate the important role of marital status in second-screening during sport games. Overall, they depict a picture of the average second-screener as a non-married older male with higher income and education, thus indicating that higher intellect combined with non-marital status, thus potentially more spare time as well as possibly higher levels of loneliness and during games are linked to sport second-screening. The results are the first to highlight the important role of marital status over young age on the tendency to second screen during sport games.

## Introduction

The nature of television viewership is rapidly changing, with surveys indicating that around 85% of viewers occasionally enhance their viewership with the use of other devices (1). This raises questions what characterizes audiences who use more than one device; for example, is it unique for young audiences and thus potentially reflects the viewership of the future, or whether it relates to education or high intellect and thus reflect elite viewing, or, alternatively, characterizes a specific gender etc. The second-screen trends are particularly evident in the sport realm, where traditional prime-time television viewing as a sole experience is largely enhanced by many sports fans with multi-platform engagement with content related to the games (2). This phenomenon, known as multi-platform browsing, involves the consumption of sports content via different devices such as smartphones, laptops/desktops, and tablets (3). Additionally, the synchronous multi-tasking of using devices during games is referred to as “second-screening” (4–6).

The aim of this paper is to contribute to a better understanding of the demographics and characteristics of individuals who enhance their sport viewership experience through second-screening and multi-platform engagement. Despite several studies conducted in the last decade (3, 7, 8), a comprehensive review of the literature from 2021 identified major gaps in understanding the demographics and motivations behind these behaviors (9).

To address these gaps, this study utilizes a case study of Israelis' TV consumption patterns during the 2022 Qatar World Cup. In our study, we tried to include a multitude of demographics in order to give a wider perspective than the one provided so far on the characteristics of second screeners. A convenience sample of 272 participants was used, and the study examined main demographic characteristics, including age, gender, income, education, and marital status. While most of these aspects were previously included in analyses (8), this study is the first to investigate the impact of marital status. Marital status is an important factor to consider due to previous studies that have found a strong association between marriage and sport engagement (10–12), mostly as a factor reducing sport consumption due to marital commitments.

The theoretical rationale behind the study is to better characterize the act of second screening and understand whether it is a reflection of intellectual enhancement of viewing, thus reflect a positive trend or whether it is related to other aspects such as boredom and loneliness, or, alternatively, is simply a mirror of young adults who are used to multi-tasking rather than focusing on the television screen. In other realms, such as political communication, there is more vibrant debate, specifically as to whether second screeners are highly involved individuals who seek to challenge the content they watch on television (13) or, alternatively, whether they mostly communicate during television viewership with like-minded individuals to reinforce their existing views and maintain their confirmation bias, thus reflecting a more negative trend (5). The analysis of the characteristics of second-screeners in the sport realm aims to advance the understanding of the potential meanings behind second screening during viewership of sport games. The study will add a unique contribution adding less studied aspects such as marital status to better understand the demographics behind second screening and thus indicate motivations behind this trend in the sport realm. The following literature review will detail the evolvement of second-screening in the sport realm and the (limited) literature (14–20) on the issue to date.

### TV sport viewing: from single task to multi-task and multi-platform

The traditional experience of watching sport games on television with undivided attention to the game is mostly associated with the radio and television glory days. However, since the first decade of the 21st century, there has been an increasing trend of enhancing TV viewing experience with multi-platform sport consumption (21). Commonly used devices for this purpose are laptops, tablets, and smartphones, which offer advanced computer abilities and web access (22). The rise of online social networks and their growing popularity has further fueled the engagement of sport fans in activities such as watching YouTube videos about teams, participating in game-related discussions on Facebook, reading tweets about the game, and following star players and athletes on Instagram before, during, and after the games (23, 24).

A 2023 YouGov analysis of nearly 20,000 sport fans from 18 countries found that young adults actually preferred watching sport games via social media broadcasting over television. However, when considering a broader perspective, television is still the preferred sport consumption platform for every other age group (22). Most studies examining the interplay between traditional TV viewing and device use during games confirm the dominant role of television in the sport consumption experience, with devices serving as secondary tools. Tang and Cooper's (3) analysis of multi-platform viewing during the 2012 Olympics, as well as Hutchins and Sanderson's (25) analysis of the 2016 Rio Games, identified that the use of digital, mobile, and social media occurred in a manner that emphasized the primary status of TV broadcasting. Tang and Cooper's (26) analysis of the viewing of the Tokyo Games further demonstrated that pre-Olympic multi-platform data consumption was the strongest predictor for watching the games on various platforms.

Despite these analyses, there have been surprisingly few studies examining the main demographics behind multi-platform sport consumption, which is a crucial aspect in understanding the main drivers behind device usage before and after games. Tang and Cooper (3) examined the impact of gender on browsing behaviors before and after sport games and found that although men and women exhibited different media consumption patterns and broader device usage during the 2008 Beijing Olympic Games, they sought similar content across various media platforms and devices. Another study by Copper and Tang (27) examined the differences in device usage between fans and non-fans during the 2008 Beijing Olympic Games, revealing similar behaviors. Regarding the role of age, as mentioned earlier, a recent YouGov study (22) unsurprisingly indicated that age plays a significant role in multi-platform adoption, with young adults being the only cohort that prefers watching games via new media devices rather than on television.

### Second screening during sport games

The phenomenon of second screening refers to the use of additional devices concurrently with television viewing, typically for activities such as reading tweets, texting about the game, and engaging in discussions about games and players on platforms like Reddit (28). One study highlighting the popularity of second screening revealed that 70% of NFL viewers use another device while watching games on TV (29). Cunningham and Eastin (2) suggest that the attractiveness and drama of games are significant factors influencing the likelihood of fans engaging in second screening. Lopez-Gonzales et al. (30) argue that the discourse on second screening underestimates its magnitude by focusing mainly on Western contexts and overlooking trends in India and China where devices are the primary platform for viewership.

Various studies have addressed the motivations behind second screening, with team identification being strongly linked to the habit. Gantz and Lewis (31) and Harboe et al. (32) found that second screening is used to foster a sense of belonging and strengthen social ties among fans. Similarly, Witkemper et al. (7) documented the role of second screening in strengthening the bond between sport fans and their teams. Phonthanukitithaworn and Sellitto (33) argue that sport fans develop and nurture a sense of community through texting during games. Billings's et al. (8) study revealed that viewers primarily used second screening for distraction and to enhance the enjoyment of the game through data collection about the game and the players.

Fewer studies have examined the demographics of second screening and the interplay between age, marital status, education, and the tendency to engage in this behavior. A study of 393 National Football League fans found that male, younger, and highly educated participants had a higher tendency to second screen Billings's et al. (8). This is, to our knowledge, the only study which highlighted a link between education and second-screening, reflecting second screening as an intellectual enhancement to television viewing. Other studies found that second screening was most prevalent among the young, with those aged 18–34 showing a similar inclination to watch games on various devices rather than solely on television (30). Similar findings were identified regarding concurrent engagement in an active mobile game while viewing a secondary screen (6, 34). Weimann-Saks et al. (35) found that in Israel, most viewers preferred to watch games together, but co-viewing did not always mean physically being in the same vicinity, as they often engaged in texting with friends during the game.

Based on the existing literature, the following hypotheses are proposed:

H1: Young adults are more likely to engage in browsing before, during, and after the game compared to other age cohorts, in line with Sim's (22) study on multi-platform consumption across generations and Billings' (8) study on second screening across different age groups.

H2: Participants with higher education are more likely to engage in browsing before, during, and after the game compared to other cohorts, in line with Billings et al., (8).

H3: Male participants are more likely to engage in browsing before, during, and after the game compared to female participants, in line with Tang and Cooper's (3) study on multi-platform sport consumption across gender and Billings's et al. (8) study on second screening across genders.

Regarding the demographic factors not extensively studied in our analysis, the following research questions are posed:

RQ1: What is the impact of income on the tendency to engage in browsing before, during, and after the game?

RQ2: What is the impact of marital status on the tendency to engage in browsing before, during, and after the game?

While the choice of the above demographic factors is in tandem with the main demographic measures in former studies of second-screeners (8) and reflects the traditional main demographic influencers examined in studies of second screening (5, 8, 33), the inclusion of marital status as a factor influencing browsing during sport games is unique to our analysis. The final section of the literature review will examine studies on the relevance of marital status in the context of sports to provide a better explanation for our decision to include this aspect.

### Marital status and sport engagement

Studies examining the effect of marriage on fitness engagement have produced mixed results. Alexandris and Carroll (36), Lee and Bhargava (37), and Bae (38) found varying outcomes in their investigations. However, a longitudinal study spanning from 1987 to 2005, which involved 8,871 adults, revealed that transitioning from singlehood to marriage was associated with an increase in fitness among women, while divorce and remarriage were linked to an increase in fitness among men (10). Conversely, a study of 3,075 adults discovered that married participants were more likely to engage in exercise compared to non-married individuals (39).

Similarly, studies investigating the effects of extreme sport challenges on married vs. non-married participants have yielded mixed results. A study of married vs. non-married ultramarathoners by Malchrowicz-Mośko and Waśkiewicz (12) found that while the challenge proved beneficial for the lives of single participants, the results were inconclusive for married participants. Additionally, a study examining the impact of marital status on the motivations of amateur marathon runners did not identify significant differences in any of the measured dimensions (40).

Regarding the impact of marital status on Tv viewing time, studies have provided more conclusive findings. Divorced, separated, and never-married adults were found to spend significantly more time watching television than married individuals, as the latter were often occupied with family-related activities (41). Furthermore, a study conducted in 2022 with a sample of 561,837 individuals found that married adults were significantly less likely to watch television for more than three hours per day compared to singles (42). Similar findings were found regarding social media (43)

Based on the above literature, our third hypothesis is proposed as follows:

H4: Married participants are less likely to consume sports via multiple platforms and engage in second screening compared to divorced and single participants.

This hypothesis is grounded in the notion that married individuals may have different priorities and responsibilities that could limit their engagement with multiple platforms and second screening during sport consumption.

## Methodology

The present study obtained ethical approval from the University Research Ethics Committee (IRB) at the first author's university in Israel. A web-based survey was used as the data collection method and was distributed among undergraduate students, as well as master's and doctoral students, enrolled in the School of Communication at a private university. Participants were encouraged to extend the distribution of the survey to their acquaintances, family members, and friends. Furthermore, data were collected via a web-based survey, which was posted on the homepages of the 3 leading sports podcasts in Israel: The Podium, Any given day, The angel.

The survey was conducted during the 2022 World Cup, specifically in November of that year, and gathered responses from a total of 272 individuals. Once the convenience sample reached 272 participants, the survey was withdrawn from the podcast homepages mentioned earlier. Participation in the survey was voluntary, and respondents were informed that their contributions would be used for statistical analysis within the framework of an academic research endeavor. Anonymity was assured, and participants were explicitly informed that their responses and decision to answer all or only a subset of the survey questions would have no implications. Participants were also notified that no incentives would be offered for their involvement and were required to acknowledge their comprehension of the terms and express agreement before proceeding with the survey. The researchers' identities, the academic institution overseeing the study, and contact information regarding the survey and collected data were disclosed.

The convenience sample consisted of 272 participants, with 43% male (Mage = 31.83, SD = 13.14). Regarding education, 34% had a high school education, 4% had some vocational studies, 38% held a BA, and 24% had a MA or PhD. Based on data from the Central Bureau of Statistics (CBS), the sampling error was ±5.95%. Distribution of gender and age was similar to CBS data with respect to the sampling error.

The survey questionnaire included approximately 46 items related to the consumption of World Cup matches across different platforms, the extent of consumption, and reasons for such consumption.

Measures: The dependent variables were “Consumption of sports on different platforms” and “Consumption of sports during World Cup games.” Participants responded on a Likert scale from 1 (not at all) to 5 (very much) regarding their usage of various platforms, including newspapers, news websites, sport websites, sport TV shows, Mondial shows, podcasts, social media, and radio. The measure of simultaneous consumption of sports content assessed participants' engagement on a scale from 1 (not at all) to 5 (very much) with activities such as watching games while browsing social networks, reading online commentary, corresponding with friends, working on the computer, working without distractions, and watching without distractions. The independent variables included gender, age, education, family status, and socio-economic status (See Appendix 1).

### Findings

Descriptive statistics: Descriptive statistics provided comprehensive demographic information about the research population, including variables such as age, gender, place of residence, education, and marital status. The results indicated that the majority of the sample population consisted of females (57%) and males (43%). The largest age group was 18–24 (43%), followed by 25–34 (27%) and 35–44 (13%). Most participants resided in the central region of the country (82%). In terms of education, the largest group held an academic degree (38%). The majority identified themselves as secular (84%). Regarding marital status, the most common status was single (52%). Data analysis showed that most participants earned an income close to or below the average (54%), while only 34% earned significantly above the average.

The survey included three key variables: “Consumption of sports on different platforms,” “Consumption of sports during World Cup games,” and “Simultaneous consumption of sports content.” Each variable was computed as the average of its constituent questions. The average score for “Consumption of sports on different platforms” was 2.00 (SD = 0.95), indicating a moderate level of consumption. The average score for “Consumption of sports during World Cup games” was 1.97 (SD = 0.93), also indicating a moderate level of consumption. Lastly, the average score for “Simultaneous consumption of sports content” was 2.09 (SD = 0.99), suggesting a moderate level of engagement in simultaneous activities while consuming sports content.

In order to examine whether there are differences in simultaneous consumption of sports content among different age groups, an ANOVA test was conducted. The results indicated a significant difference among the age groups [*F* (6, 262) = 7.416, *p* < 0.001]. Specifically, participants in the 18–24 age group exhibited the lowest average level of simultaneous consumption of sports content (*M* = 1.71, SD = 0.95), while individuals in the 35–44 age group demonstrated the highest average level (*M* = 2.78, SD = 0.62). (See, Table 1).

**Table 1:** The variable, consumption of sports during world cup matches.

Descriptives									
		*N*	Mean	Std. deviation	Std. error	95% confidence interval for mean		Minimum	Maximum
						Lower bound	Upper bound		
Simultaneous consumption of sports content	Under then 18	1	2.67					3	3
	18–24	114	1.71	0.95	0.09	1.54	1.89	1	5
	25–34	74	2.23	1.05	0.12	1.98	2.47	1	5
	35–44	35	2.78	0.62	0.11	2.56	2.99	1	4
	45–54	24	2.45	0.80	0.16	2.11	2.79	1	4
	55–64	12	2.06	0.86	0.25	1.51	2.60	1	4
	Up then 65	9	2.07	0.67	0.22	1.56	2.59	2	4
	Total	269	2.09	0.98	0.06	1.97	2.21	1	5

**Table:** 

ANOVA						
		Sum of squares	df	Mean square	*F*	Sig.
Simultaneous consumption of sports content	Between Groups	37.656	6.00	6.276	7.416	0.000
	Within Groups	221.730	262.00	0.846		
	Total	259.386	268.00			

ANOVA Test. Dependent Variable: Simultaneous consumption of sports content. Independent Variable: Age groups.

### Testing for significant differences of the research variables according to family status

In order to examine whether there are differences in sports consumption across different family statuses on various platforms, an independent samples *t*-test was conducted. The results revealed that sports consumption across different platforms among single/divorced individuals (*M* = 2.25, SD = 0.93) was significantly higher than among married individuals (*M* = 1.70, SD = 0.91). The *t*-test analysis yielded a significant *t*-value of −4.913 (df = 273, *p* < 0.001), indicating a substantial difference between the two groups. (See, Table 2).

**Table 2:** Variable, “sports consumption on different platforms”.

Married		*N*	Mean	Std. deviation	Std. error mean
Sports consumption on different platforms	married	125	1.7056	0.90576	0.08101
	single	150	2.2513	0.92664	0.07566

**Table:** 

Independent samples test										
		Levene's test for equality of variances		*t*-test for equality of means						
		*F*	Sig.	*t*	df	Sig. (2-tailed)	Mean difference	Std. error difference	95% confidence interval of the difference	
									Lower	Upper
Sports consumption on different platforms	Equal variances assumed	0.219	0.640	−4.913	273	*p* < 0.01	−0.54573	0.11108	−0.76441	−0.32704
	Equal variances not assumed			−4.923	266.143	*p* < 0.01	−0.54573	0.11085	−0.76398	−0.32747

Independent *T* Test. Dependent Variable: Sports consumption across different platforms. Independent Variable: Family Status.

To examine whether there are differences in sports consumption during the World Cup games based on family status, an independent samples-test was conducted. The results revealed that sports consumption during world cup matches among single/divorced individuals (*M* = 2.21, SD = 0.91) was significantly higher than among married individuals (*M* = 1.68, SD = 0.87). The *t*-test analysis yielded a significant *t*-value of −4.887 (df = 272, *p* < 0.001), indicating a substantial difference between the two groups. (See, Table 3).

**Table 3:** Variable: “sports consumption during world cup matches”.

Group statistics					
married		*N*	Mean	Std. deviation	Std. error mean
Sports consumption during world cup matches	married	125	1.6843	0.86764	0.07760
	single/divorced	149	2.2139	0.91457	0.07492

**Table:** 

Independent samples test										
		Levene's test for equality of variances		*t*-test for equality of means						
		*F*	Sig.	*t*	df	Sig. (2-tailed)	Mean difference	Std. error difference	95% Confidence interval of the difference	
									Lower	Upper
Sports consumption during world cup matches	Equal variances assumed	0.064	0.801	−4.887	272	*p* < 0.01	−0.52961	0.10837	−0.74296	−0.31625
	Equal variances not assumed			−4.910	267.891	*p* < 0.01	−0.52961	0.10787	−0.74199	−0.31722

Independent *T* Test. Dependent Variable: Sports consumption during World Cup games. Independent Variable: Family Status.

In order to examine whether there are differences in simultaneous consumption of sports during the World Cup matches based on family status, an independent samples *t*-test was conducted. The results revealed that simultaneous consumption of sports during the World Cup among single/divorced individuals (*M* = 2.32, SD = 0.96) was significantly higher than among married individuals (*M* = 1.84, SD = 0.97). The *t*-test analysis yielded a significant *t*-value of −3.924 (df = 270, *p* < 0.001), indicating a substantial difference between the two groups. (See, Table 4).

**Table 4:** Variable: “simultaneous sports content consumption”.

Group statistics					
Married		*N*	Mean	Std. deviation	Std. error mean
Simultaneous sports content consumption	married	123	1.8412	0.96941	0.08741
	single/divorced	149	2.3029	0.96302	0.07889

**Table:** 

Independent samples test										
		Levene's test for equality of variances		*t*-test for equality of means						
		*F*	Sig.	*t*	df	Sig. (2-tailed)	Mean difference	Std. error difference	95% Confidence interval of the difference	
									Lower	Upper
Simultaneous sports content consumption	Equal variances assumed	0.856	0.356	−3.924	270	*p* < 0.01	−0.46172	0.11767	−0.69339	−0.23004
	Equal variances not assumed			−3.921	259.680	*p* < 0.01	−0.46172	0.11775	−0.69358	−0.22985

In order to examine whether there are differences in viewing behavior during a game based on family status, an independent samples *t*-test was conducted. The results revealed that viewing behavior, (“watching with one friend” to “watching with a group of friends”), during a game among married individuals (*M* = 1.93, SD = 0.944) was significantly higher than among single/divorced individuals (*M* = 1.28, SD = 1). The *t*-test analysis yielded a significant *t*-value of 5.418 (df = 253.150, *p* < 0.001), indicating a substantial difference between the two groups. (See, Table 5).

**Table 5:** The variable “game viewing”.

Group statistics					
Married		*N*	Mean	Std. deviation	Std. error mean
Game viewing	married	117	1.93	0.944	0.087
	single/divorced	144	1.28	1.000	0.083

**Table:** 

Independent samples test										
		Levene's test for equality of variances		*t*-test for equality of means						
		*F*	Sig.	*t*	df	Sig. (2-tailed)	Mean difference	Std. error difference	95% Confidence interval of the difference	
									Lower	Upper
Game viewing	Equal variances assumed	5.867	0.016	5.387	259	*p* < 0.01	0.654	0.121	0.415	0.893
	Equal variances not assumed			5.418	253.150	*p* < 0.01	0.654	0.121	0.416	0.891

Independent *T* Test. Dependent Variable:” game viewing”. Independent Variable: Family Status.

### Testing for significant differences of the research variables according to socioeconomic status

In order to examine whether there are differences in sports consumption on different platforms according to income level, an ANOVA test was performed. The results showed a significant difference between the different types of income levels [*F* (4, 270) = 8.684, *p* < 0.001]. Specifically, the analysis revealed that individuals earning slightly above average reported the highest consumption of sports across platforms (*M* = 2.57, SD = 0.89), while individuals earning slightly below average reported the lowest level of consumption of sports across platforms (*M* = 1.74, SD = 0.98). (See Table 6).

**Table 6:** Variable “sports consumption on different platforms”.

		*N*	Mean	Std. deviation	Std. error	95% Confidence interval for mean		Minimum	Maximum
						Lower bound	Upper bound		
Sports consumption on different platforms	Below average	66	1.76	0.93	0.11486	1.5330	1.9918	1.00	4.33
	Slightly below average	82	1.74	0.98	0.10791	1.5246	1.9540	1.00	4.58
	Average	32	1.91	0.80	0.14067	1.6250	2.1988	1.00	3.83
	Slightly above average	42	2.57	0.89	0.13729	2.2907	2.8452	1.00	4.42
	Well above average	53	2.32	0.83	0.11342	2.0916	2.5468	1.00	4.00
	Total	275	2.00	0.96	0.05760	1.8899	2.1166	1.00	4.58

**Table:** 

ANOVA						
		Sum of squares	df	Mean square	*F*	Sig.
Sports consumption on different platforms	Between Groups	28.493	4	7.123	8.684	0.000
	Within Groups	221.482	270	0.820		
	Total	249.976	274			

ANOVA Test. Dependent Variable: Sports consumption on different platforms. Independent Variable: Income Level.

In order to examine whether there are differences in sports consumption during the World Cup games based on income levels, an ANOVA test was conducted. The results revealed a significant difference among the different income levels [*F* (4, 269) = 7.659, p < 0.001]. Specifically, the analysis revealed that individuals with slightly above-average income reported the highest level of sports consumption during the World Cup games (*M* = 2.48, SD = 0.87), while individuals with much lower income reported the lowest level (*M* = 1.70, SD = 0.85). (See Table 7).

**Table 7:** The variable, *consumption of sports during world cup matches*.

		*N*	Mean	Std. deviation	Std. error	95% Confidence interval for mean		Minimum	Maximum
						Lower bound	Upper bound		
Consumption of sports during world cup matches	Below average	65	1.70	0.85	0.10602	1.4864	1.9101	1.00	4.17
	Slightly below average	82	1.76	0.97	0.10683	1.5464	1.9716	1.00	4.75
	Average	32	1.92	0.81	0.14393	1.6263	2.2133	1.00	3.92
	Slightly above average	42	2.48	0.87	0.13477	2.2078	2.7521	1.00	4.36
	Well above average	53	2.27	0.85	0.11689	2.0332	2.5023	1.00	4.00
	Total	274	1.97	0.93	0.05619	1.8617	2.0829	1.00	4.75

**Table:** 

ANOVA						
		Sum of squares	df	Mean square	*F*	Sig.
Consumption of sports during world cup matches	Between Groups	24.151	4	6.038	7.659	0.000
	Within Groups	212.056	269	0.788		
	Total	236.207	273			

ANOVA Test. Dependent Variable: consumption of sports during World Cup matches. Independent Variable: Income Level.

In order to examine whether there are significant differences in parallel consumption of sports-related content based on income level, an ANOVA test was conducted. The results revealed a significant difference among the different income levels [*F* (4, 269) = 7.659, *p* < 0.001]. Specifically, the analysis revealed that individuals with slightly above-average income reported the highest level of parallel consumption of sports-related content (*M* = 2.67, SD = 0.78), while individuals with significantly lower income reported the lowest level (*M* = 1.78, SD = 1.00). (See Table 8).

**Table 8:** Variable: “simultaneous sports content consumption”.

		*N*	Mean	Std. deviation	Std. error	95% Confidence interval for mean		Minimum	Maximum
						Lower bound	Upper bound		
Simultaneous sports content consumption	Below average	63	1.78	1.00	0.12608	1.5310	2.0351	1.00	4.67
	Slightly below average	82	1.93	1.03	0.11330	1.7087	2.1596	1.00	4.67
	Average	32	1.95	0.87	0.15396	1.6391	2.2671	1.00	3.83
	Slightly above average	42	2.67	0.78	0.11968	2.4250	2.9084	1.00	4.00
	Well above average	53	2.34	0.93	0.12758	2.0868	2.5988	1.00	4.50
	Total	272	2.09	0.99	0.06010	1.9758	2.2124	1.00	4.67

**Table:** 

ANOVA						
		Sum of squares	df	Mean square	*F*	Sig.
Simultaneous sports content consumption	Between Groups	24.151	4	6.038	7.659	0.000
	Within Groups	212.056	269	0.788		
	Total	236.207	273			

ANOVA Test. Dependent Variable: Simultaneous Sports Content Consumption. Independent Variable: Income Level.

### Testing for significant differences of the research variables according to the level of education

In order to examine whether there are differences in sports consumption across different educational levels, an ANOVA test was conducted. The results revealed a significant difference among the various levels of educational attainment [*F* (3, 272) = 16.491, *p* < 0.001]. Specifically, the analysis indicated that individuals with postgraduate or doctoral degrees reported the highest level of sports consumption across different platforms (*M* = 2.63, SD = 0.72), while individuals with only a secondary education reported the lowest level of sports consumption (*M* = 1.63, SD = 0.92). (See Table 9).

**Table 9:** Variable, sports consumption on different platforms.

		*N*	Mean	Std. deviation	Std. error	95% Confidence interval for mean		Minimum	Maximum
						Lower bound	Upper bound		
Sports consumption on different platforms	HI SCHOLL	95	1.63	0.92	0.09398	1.4473	1.8205	1.00	4.58
	Post intuition	11	2.02	0.82	0.24776	1.4645	2.5686	1.00	3.17
	Academic	105	1.95	0.95	0.09231	1.7702	2.1363	1.00	4.50
	Master's-PhD	65	2.63	0.72	0.08895	2.4490	2.8044	1.00	4.42
	Total	276	2.00	0.95	0.05740	1.8914	2.1174	1.00	4.58

**Table:** 

ANOVA						
		Sum of squares	df	Mean square	*F*	Sig.
Sports consumption on different platforms	Between Groups	38.487	3	12.829	16.491	0.000
	Within Groups	211.597	272	0.778		
	Total	250.084	275			

ANOVA Test. Dependent Variable: sports consumption on different platforms. Independent Variable: Educational Level.

In order to examine whether there are differences in sports consumption during the World Cup matches based on educational attainment, an ANOVA test was conducted. The results revealed a significant difference among the different levels of education [*F* (3, 271) = 15.330, *p* < 0.001]. Specifically, the analysis showed that individuals with a master's or doctoral degree reported the highest level of sports consumption during the World Cup matches (*M* = 2.54, SD = 0.74), while individuals with a moderate level of education reported the lowest level of sports consumption (*M* = 1.60, SD = 0.87). (See Table 10).

**Table 10:** The variable, *consumption of sports during world cup matches*.

		*N*	Mean	Std. deviation	Std. error	95% Confidence interval for mean		Minimum	Maximum
						Lower bound	Upper bound		
Consumption of sports during world cup matches	HI SCHOLL	95	1.60	0.87	0.08935	1.4191	1.7739	1.00	4.75
	Post intuition	11	1.93	0.71	0.21523	1.4543	2.4135	1.00	2.83
	Academic	105	1.97	0.93	0.09109	1.7873	2.1486	1.00	4.25
	Master's-PhD	64	2.54	0.74	0.09310	2.3568	2.7289	1.00	4.36
	Total	275	1.97	0.93	0.05599	1.8618	2.0823	1.00	4.75

**Table:** 

ANOVA						
		Sum of squares	df	Mean square	*F*	Sig.
Consumption of sports during world cup matches	Between Groups	34.270	3	11.423	15.330	0.000
	Within Groups	201.941	271	0.745		
	Total	236.211	274			

ANOVA Test. Dependent Variable: consumption of sports during World Cup matches. Independent Variable: Educational Level.

In order to examine whether there are differences in Simultaneous Sports Content Consumption based on educational attainment, an ANOVA test was conducted. The results revealed a significant difference among the different levels of education [*F* (3, 269) = 11.018, *p* < 0.001]. Specifically, the analysis indicated that individuals with a postgraduate degree reported the highest level of Simultaneous Sports Content Consumption (*M* = 2.58, SD = 0.70), whereas individuals with a moderate level of education reported the lowest level of Simultaneous Sports Content Consumption (*M* = 1.72, SD = 0.97). (See Table 11).

**Table 11:** Variable: “simultaneous sports content consumption”.

		*N*	Mean	Std. deviation	Std. error	95% Confidence interval for mean		Minimum	Maximum
						Lower bound	Upper bound		
Simultaneous sports content consumption	HI SCHOLL	95	1.72	0.97	0.09938	1.5237	1.9184	1.00	4.67
	Post intuition	11	1.92	0.81	0.24450	1.3795	2.4690	1.00	3.33
	Academic	103	2.15	1.04	0.10293	1.9441	2.3524	1.00	4.50
	Master's-PhD	64	2.58	0.70	0.08800	2.4075	2.7592	1.00	4.00
	Total	273	2.09	0.99	0.05990	1.9746	2.2105	1.00	4.67

**Table:** 

ANOVA						
		Sum of squares	df	Mean square	*F*	Sig.
Simultaneous sports content consumption	Between Groups	29.157	3	9.719	11.018	0.000
	Within Groups	237.295	269	0.882		
	Total	266.453	272			

ANOVA Test. Dependent Simultaneous Sports Content Consumption. Independent Variable: Educational Level.

### Testing for significant differences of the research variables according to the participants' gender

To examine whether there are differences in average sports consumption across different platforms based on gender, an independent samples *t*-test was conducted. The results revealed that sports consumption on different platforms among males (*M* = 2.67, SD = 0.87) was significantly higher than among females (*M* = 1.48, SD = 0.65). The *t*-test analysis yielded a significant *t*-value of 12.356 (df = 204.490, *p* < 0.001), indicating a substantial difference between the two groups. (See Table 12).

**Table 12:** Variable, sports consumption on different platforms.

Group statistics					
Gender		*N*	Mean	Std. deviation	Std. error mean
Sports consumption on different platforms	Male	116	2.67	0.87	0.081
	Female	156	1.48	0.65	0.052

**Table:** 

Independent samples test										
		Levene's test for equality of variances		*t*-test for equality of means						
		*F*	Sig.	*t*	df	Sig. (2-tailed)	Mean difference	Std. error difference	95% Confidence interval of the difference	
									Lower	Upper
Sports consumption on different platforms	Equal variances assumed	15.202	0.000	12.881	270	*p* < 0.01	1.185	0.092	1.004	1.366
	Equal variances not assumed			12.356	204.490	*p* < 0.01	1.185	0.096	0.996	1.374

Independent *T* Test. Dependent Variable: sports consumption on different platforms. Independent Variable: Gender.

To examine whether there are differences in sports consumption during the World Cup matches based on gender, an independent samples *t*-test was conducted. The results revealed that sports consumption across different platforms among males (*M* = 2.60, SD = 0.84) was significantly higher than among females (*M* = 1.49, SD = 0.66). The *t*-test analysis yielded a significant *t*-value of 11.720 (df = 210.302, *p* < 0.001), indicating a substantial difference between the two groups. (See, Table 13).

**Table 13:** The variable, sports consumption during the world cup matches.

Group statistics					
Gender		*N*	Mean	Std. deviation	Std. error mean
Sports consumption during the world cup matches	Male	115	2.60	0.84	0.078
	Female	156	1.49	0.66	0.053

**Table:** 

Independent samples test											
		Levene's test for equality of variances		*t*-test for equality of means							
		*F*	Sig.	*t*	df	Significance		Mean difference	Std. error difference	95% Confidence interval of the difference	
						One-sided *p*	Two-sided *p*			Lower	Upper
Sports consumption during the world cup matches	Equal variances assumed	11.052	0.001	12.139	269	*p* < 0.01	*p* < 0.01	1.111	0.092	0.931	1.291
	Equal variances not assumed			11.720	210.302	*p* < 0.01	*p* < 0.01	1.111	0.095	0.924	1.298

Independent *T* Test. Dependent Variable: sports consumption during the World Cup matches. Independent Variable: Gender.

To investigate whether there are differences in parallel sports content consumption based on gender, an independent samples *t*-test was conducted. The results revealed no significant differences in sports consumption across different platforms between males and females.

## Discussion and conclusions

The present study aimed to examine the use of multi-platform and second-screen consumption during sport events and understand the demographics behind these trends. The analysis revealed several interesting findings, with the most notable being the tendency for marriage to reduce the likelihood of browsing before, during, and after games. Single and divorced participants showed a significantly higher inclination to consume information from multiple platforms before and after games, moreover single and divorced participants were more likely to engage in second-screening during games. In contrast, married participants exhibited the lowest levels of both multi-platform browsing and second-screening.

One possible explanation for these findings is that the survey found that married individuals often watch games in the company of others, whereas single and divorced individuals, who tend to consume a variety of media, including television and social media, have more time and inclination to browse before, during, and after sport games. Further, it sits with the tendency of married individuals to reduce engagement with physical sport and sport viewership vs. single and divorced adults' tendency to spend a lot more time than married watching television (42). Future studies could explore the role of boredom, spare time, and attempts to alleviate loneliness in the browsing habits of divorced and single individuals, as previous research has linked these factors to higher levels of loneliness and spare time in comparison to married adults.

Another noteworthy finding was that young adults were less likely than other age groups to engage in second-screening. Interestingly, young adults reported watching the games more often with friends or in public places. This finding supports a previous study conducted in Israel, which also highlighted the high tendency of Israelis to watch games together, emphasizing the importance of cultural factors and a sense of togetherness in the use of devices beyond television during games (35); see also (44). While distraction has been identified as a major motivation for second-screening (8), the presence of people around young adult Israelis may reduce the need for distraction, providing a possible explanation for this unique result. Future studies should further explore the interplay between culture and the tendency to browse for information during sport games to gain a better understanding of their relationship.

The study's other results align with existing literature. men reported higher levels of browsing before and after games compared to female participants, although no significant differences were found in reported levels of second-screening during games. These findings are consistent with previous research, which has shown mixed results regarding gender differences in multi-platform and second-screen usage during sports events (3, 8). Additionally, the study found a positive association between higher education and the propensity to browse around games, aligning with previous research on the profile of second-screen users and the link between education and interest in sports information (8, 35). Similarly, while there are no specific studies on the interplay between income and second-screening, the study identified a higher tendency for individuals with higher income to engage in browsing before, during, and after games (45, 46). These findings align with wider literature linking sports engagement and income (Ibid.).

Overall, the study paints a profile of a typical second-screener who also engages in browsing before and after games as a non-married older male with higher income and education. It is likely that individuals with this profile have more spare time, experience boredom and loneliness, and have an interest in sports data that aligns with their education and income (8, 35, 46, 47).

Furthermore, the findings indicate that individuals with higher educational attainment and higher income levels demonstrate a propensity to actively seek information before, during, and after engaging in sports games. These findings align with existing research on information consumption and the consumption of in-depth content via podcast platforms (8, 45), suggesting that individuals with higher education and income have a greater inclination to consume information in various contexts, including sports games.

In conclusion, this study provides valuable insights into the demographics of multi-platform and second-screen usage during sport events. The findings suggest that marital status, age, gender, education, and income are important factors influencing these consumption behaviors. Divorced and single individuals, as well as those with higher education and income, are more likely to engage in multi-platform browsing and second-screening during sport games. Young adults, on the other hand, tend to watch games with friends or in public places, which may reduce their need for second-screen engagement. The results make a unique contribution to existing studies as they are the first to highlight the importance of marital status in the tendency to use a second screen during sport games. Whereas so far this aspect was mostly ignored in second screen studies, our analysis indicates that it is more important than other components which were indicated in former studies, such as the young adults age group. One possible explanation might be the Israeli culture where a sense of togetherness encourages watching games toger and as noted above, we hope that future comparative studies will address this issue, These findings contribute to a better understanding of the motivations and characteristics of individuals who enhance their sport viewership experience through the use of multiple devices and platforms.

It is important to note that the study has certain limitations, such as the use of a convenience sample and the potential for sampling bias. Convenience sampling offers rapid data collection, ease of access, and practical feasibility, making it particularly advantageous for quick insights and exploratory research. However, it is important to acknowledge that convenience sampling may introduce bias which should be considered when interpreting findings. The sample primarily consisted of MA and BA students from a school of communication and sports podcast consumers, resulting in an overrepresentation of female participants (57%) and the 18–24 age group (43%), thereby limiting the external validity of the findings.

Future research should aim to address these limitations by employing more representative samples and exploring additional factors that may influence multi-platform and second-screen consumption during sport events. Nonetheless, this study provides valuable insights into the changing nature of television viewership and the increasing prevalence of multi-platform and second-screen engagement in the context of sport games.

## Data Availability

The original contributions presented in the study are included in the article/Supplementary Material, further inquiries can be directed to the corresponding author.

## Appendix

### Appendix 1 - Survey questions

How do you define your gender identity?

• Male
• Female

Place of residence:

• North
• Central
• South
• Other

Education:

• High school
• Post-high school
• Academic
• Second or third degree

Religious affiliation:

• Secular
• Traditional
• Religious
• Ultra-Orthodox

Marital status:

• Single
• Married or in a relationship
• Divorced
• Widowed

The average income per person in a household is 9,000 NIS gross per month. How is your income in compare?

• Much below average
• Below average
• Average
• Slightly above average
• Much above average

Please specify the extent to which you consume sports content on the following platforms:

**Table:**

Extremely	Very	Moderately	Slightly	Not at all
Read sports sections in newspapers				
Keep up with sports news websites				
Browse dedicated sports websites				
Watch live sports broadcasts on television				
Watch sports programs on television (e.g., sports news)				
Listen to sports podcasts in Hebrew				
Listen to sports podcasts in a foreign language				
Watch sports content on YouTube or similar platforms				
Keeps up with sports content on social media networks				
Listen to sports programs on the radio				
Follow on Twitter (but doesn't watch)				
Follow on TikTok (but doesn't watch)				

Please specify the extent to which you consume sports content during World Cup games (November–Decemeber 2022) on the following platforms:.

**Table:**

Extremely	Very	Moderately	Slightly	Not at all
Read sports sections in newspapers				
Keep up with sports news websites				
Browse dedicated sports websites				
Watch live sports broadcasts on television				
Watch sports programs on television (e.g., sports news)				
Listen to sports podcasts in Hebrew				
Listen to sports podcasts in a foreign language				
Watch sports content on YouTube or similar platforms				
Keeps up with sports content on social media networks				
Listen to sports programs on the radio				
Follow on Twitter (but doesn't watch)				
Follow on TikTok (but doesn't watch)				

Please specify to what extent you simultaneously integrate World Cup content across the following platforms:.

**Table:**

Extremely	Very	Moderately	Slightly	Not at all
I watch the game and browse social media				
I watch the games and only read online commentary.				
I watch the games and chat with friends.				
I watch the games while working on the computer.				
I watch the games without any distractions.				
I follow the game online while working				

How many hours do you dedicate to watching World Cup games?.

• Less than an hour per day (prefer to watch daily summaries).
• Between one to two hours.
• Between three to five hours.
• More than five hours.
• More than seven hours.

Preferred viewing arrangement:.

• I prefer to watch alone.
• I watch with one friend.
• I watch with a group of friends.
• Prefer to go to a place with communal viewing (pub, park, etc.).
